# A 50-week walking intervention for type 2 diabetes mellitus: A pilot study to improve fitness, BMI, and quality of life outcomes

**DOI:** 10.1016/j.pmedr.2025.103299

**Published:** 2025-11-01

**Authors:** Jesper Mulder, Anne K. Smit, Mirte Boelens, Jessica C. Kiefte-de Jong

**Affiliations:** a Health Campus The Hague, Department of Public Health and Primary Care, Leiden University Medical Center, The Hague, the Netherlands; b Department of Human Movement Sciences, University Medical Centre Groningen, Groningen, the Netherlands

**Keywords:** Diabetes, Physical fitness, Wellbeing, Lifestyle, Group intervention

## Abstract

**Objective:**

This study investigated whether a multicomponent 50-week walking intervention, including guidance from lifestyle professionals, improved physical fitness, body mass index (BMI), and quality of life (QoL).

**Methods:**

Forty-five Dutch individuals with or at high risk of type 2 diabetes mellitus (T2DM) participated in this pilot study between April 2023 and March 2024. Total intervention duration was 50 weeks, of which 30 weeks with guidance and 20 weeks without guidance. Physical fitness (6MWT), BMI, and QoL were assessed at week 0 (T0), 30 (T1), and 50 (T2). The experience of participants and professionals (*n* = 17) was also inventoried.

**Results:**

Linear mixed models on all 45 participants indicated no significant changes over time. Participants with complete data (*n* = 12) indicated a significant change in EQ-VAS (β = 4.792, CI = 1.439, 8.145) over time, while 6MWT, BMI, and EQ-index did not change.

**Conclusion:**

This pilot study elicited positive responses by participants and professionals. However, adherence to the intervention could not be assessed based on the questionnaire responses. A multicomponent walking intervention with lifestyle coaching could be used to optimize lifestyle interventions in T2DM, although the current study showed that individual differences, motivation, and adherence to changes should be considered.

## 1 Introduction

Over 800 million people are suffering from type 2 diabetes (T2DM) (World Health Organization, 2024), and about 15 % of the global population is at risk (i.e., suffering from prediabetes, defined as impaired glucose tolerance or fasting glucose) (Rooney et al., 2023). T2DM is a noncommunicable disease that can be managed through lifestyle interventions and, if needed, glucose-lowering medication. A key lifestyle factor that is associated with increased risk of T2DM is physical inactivity (Biswas et al., 2015; Diabetes Prevention Program Research G et al., 2009; World Health Organization, 2021), especially since, according to global estimates by the World Health Organization (WHO), approximately 28 % of adults do not meet sufficient levels of physical activity (World Health Organization, 2021).

People suffering from or at risk of T2DM may experience several barriers for participation in physical activity, but walking may be an accessible and feasible type of physical activity to improve overall health. Since lack of social support and confidence are key barriers to physical activity for those with at risk of T2DM, walking interventions may also benefit from focusing on peer support and confidence-building elements. Additionally, addressing other lifestyle factors alongside increased physical activity may be beneficial for those with or at increased risk of T2DM.

Besides improving parameters of physical fitness and other lifestyle factors, multicomponent interventions can also improve QoL in people suffering from or at risk of T2DM (Jing et al., 2018). Therefore, the current study investigates whether a 50-week multicomponent walking intervention, including professional guidance, improves physical fitness levels, QoL, and BMI in this population.

## 2 Methods

### 2.1 Population and study design

Participants were individuals suffering from or at risk of T2DM, as diagnosed by physicians. All participants were recruited in Hilversum, a municipality in the Netherlands with about 94,000 inhabitants, by professionals (e.g., general practitioners, community sports coaches, dieticians, lifestyle coaches) and (digital) advertisement. Initial recruitment included 37 participants who completed baseline assessment (T0). Recruitment was open until T1, thus between T0 and T1 an additional 8 participants were enrolled into the study, resulting in 45 participants at T1. This pilot study was conducted under the broader Diabetes Challenge project, which received ethical approval (METC number: 19–035).

The ‘Diabetes in Beweging’ (‘Diabetes on the move’ in Dutch) intervention is a 50-week multicomponent walking intervention aimed at individuals diagnosed with T2DM and individuals at risk of T2DM, developed by the Bas van de Goor Foundation (Diabetes in Beweging, 2025), taking place between April 2023 and March 2024. The intervention is divided into five blocks of 10 weeks, with different lifestyle themes (Appendix A). During the first three blocks (30 weeks) participants walked in groups accompanied by professionals (i.e., dieticians, sports-, and lifestyle coaches). During the final two blocks (20 weeks) participants walked on their own or with other participants. During all blocks there was online coaching available. Participants walked once every week, which included several low intensity muscle strength and coordination exercises. Participants walked at their regular walking pace, for about an hour. The primary goal of the intervention was to improve physical fitness, which in turn improves BMI and QoL.

### 2.2 Outcome measures

Participants were measured at week 1 (T0), week 30 (T1), and week 50 (T2). Participants completed a six-minute walking test (6MWT) on a flat surfaced 50-m course to assess physical fitness (Crapo et al., 2002). Number of completed laps was noted and multiplied by 50 to calculate total distance completed. Participants were instructed to walk at a comfortable pace (i.e., their regular walking pace) and refrain from talking during the test.

BMI (kg/m^2^) was calculated based on self-reported height and measured weight. Weight was measured without shoes and sweaters/vests. BMI was computed as weight in kilograms divided by height in meters squared.

QoL was assessed with the EuroQol 5-dimensional instrument (EQ-5D-5L) (Stolk et al., 2019; Versteegh et al., 2016), a validated instrument that measures QoL in five dimensions (mobility, self-care, usual activities, pain/discomfort, and anxiety/depression). Dimensions are rated on a 5-point Likert scale, from no problems (1) to extreme problems (5), with a lower score indicating a better score. Applying utility weights across the five dimensions, a summary index value (EQ-index) range relative to full health (index value 1) and death (index value 0) is derived. The EQ-5D-5L also includes perceived health on a Visual Analogue Scale (VAS) ranging from 0 to 100 with a higher score indicating a better QoL. We used both the summary index value (EQ-index) and the VAS (EQ-VAS) to evaluate QoL.

### 2.3 Covariates

age, years of diabetes, total active minutes per week, sex, education level, migration background, presence of diabetes and multimorbidity, and whether Dutch physical activity guidelines were met. Participants filled out an online questionnaire at baseline including questions about their sociodemographic data. All participants received an online questionnaire at T2 via e-mail, to evaluate program satisfaction on a 0–10 scale, and providing qualitative feedback through open text.

### 2.4 Statistical analyses

For the outcome measures, outliers >3SD were removed. Data for the EQ-index and EQ-VAS were considered non-normally distributed and transformed using natural logarithmic transformations. The 6MWT and BMI data were normally distributed. To examine 6MWT, BMI, and QoL changes over time, linear mixed model analyses were performed on all available datapoints even if data was missing, since these analyses are robust to missing data at timepoints and thus ensuring efficient use of available data. Missing data was not imputed as previous studies showed that imputation can be unstable in longitudinal analyses (Twisk et al., 2013). Analyses were not adjusted for covariates, for various reasons: 1) covariates were stable over time, 2) participants were their own controls, and 3) the study was not powered to include covariates in the regression analyses. Sensitivity analyses including participants with two or more measures and complete data were performed (Appendix B). Custom Python (version 3.10) scripts were used for data analysis. All hypotheses were evaluated with an alpha of 0.05 (two-sided).

## 3 Results

Participants of this pilot study had a mean age ranging from 64.4 to 66.7 years across all timepoints (Table 1). They were characterized by being mainly female, lower educated, and with no migration background. Most participants did not meet Dutch physical activity guidelines. Presence of diabetes and multimorbidity was equally distributed.

**Table 1:** Descriptive statistics of 45 Dutch adults with or at risk of type 2 diabetes mellitus at week 1 (T0), week 30 (T1), and week 50 (T2). Data collection took place between April 2023 and March 2024.

	T0 (*n* = 37 [82 %])	T1 (*n* = 45 [100 %])	T2 (*n* = 22 [49 %])
Continuous variables (M (SD) or Md (25 % IQR, 75 % IQR))			
Age	64.4 (11.4)	64.7 (10.7)	66.7 (8.1)
Years of diabetes	10 (4.5, 18)^a^	10 (4.3, 19)^c^	10 (7, 15.3)^g^
Total active minutes per week	90 (50, 150)	60 (0, 125)	110 (63.8, 150)
Six-minute walking test (meters)	486.5 (52.7)^b^	467.1 (61.8)^d^	492.6 (66.1)^h^
BMI	31.7 (5.7)^b^	30.3 (5.3)^e^	29.6 (4.8)^b^
EQ-index	0.85 (0.77, 0.91)	0.85 (0.74, 1)^f^	0.86 (0.81, 0.92)^i^
EQ-VAS	75 (60, 85)	80 (68.8, 90)^f^	110 (63.8, 150)

Categorical variables (frequency and percentage)			
Sex			
Female	28 (75.7)	33 (73.3)	15 (68.2)
Male	9 (24.3)	12 (26.7)	7 (31.8)
Education level			
Low	25 (69.4)	31 (70.5)	17 (81.0)
High	11 (60.6)	13 (29.5)	4 (19.0)
Migration background			
No migration background	31 (86.1)	38 (86.4)	19 (90.5)
With migration background	5 (13.9)	6 (13.6)	2 (9.5)
Diabetes			
No	17 (46.0)	21 (46.7)	7 (31.8)
Yes	20 (54.0)	24 (53.3)	15 (68.2)
Multimorbidity			
No	18 (48.7)	21 (46.7)	10 (45.5)
Yes	19 (51.3)	24 (53.3)	12 (54.5)
Meets PA guidelines (>150 min/week)			
No	27 (73.0)	35 (77.8)	14 (63.6)
Yes	10 (27.0)	10 (22.2)	8 (36.4)

M = Mean, SD = Standard Deviation, Mdn = Median, IQR = Interquartile Range. Note: 8 participants were not assessed at T0 and entered the intervention at T1; from T1 to T2 23 participants dropped out.

a 9 missing values.

b 1 missing value.

c 23 missing values.

d 19 missing values.

e 14 missing values.

f 17 missing values.

g 8 missing values.

h 7 missing values.

i 2 missing values

Linear mixed model analyses (Table 2) indicated no significant changes over time in 6MWT (β = −3.873, CI = −15.304, 7.559), BMI (β = −0.152, CI = −0.403, 0.100), EQ-index (β = −0.009, CI = 2.800, 0.025), and EQ-VAS (β = 0.022, CI = 0.377, 3.049). Sensitivity analyses using participants with two or more timepoints revealed no significant changes over time in 6MWT, BMI, EQ-index, or EQ-VAS. Analyses limited to participants with complete data only showed a significant change over time for EQ-VAS in the complete-case sample (β = 4.792, CI = 1.439, 8.145; Appendix B).

**Table 2:** Linear mixed model analyses results of the six-minute walking test (6MWT), body mass index (BMI), quality of life index (EQ-index), and quality of life visual analogue scale (EQ-VAS) outcome measures in 45 Dutch adults with or at high risk of type 2 diabetes mellitus over three timepoints (week 0, week 30, and week 50). Data collection took place between April 2023 and March 2024.

	β	CI Lower	CI Upper
*6MWT*			
Time	−3.873	−15.304	7.559
Group	1.843	0.267	3.419
*BMI*			
Time	−0.152	−0.403	0.100
Group	47.97	18.836	77.104
*EQ-index*			
Time	−0.009	2.800	0.025
Group	8.776	4.102	14.752
*EQ-VAS*			
Time	0.022	−0.036	0.080
Group	1.713	0.377	3.049

Note: Linear mixed model analyses were not adjusted for covariates.

Seventeen participants graded the program as satisfactory, with scores ranging from 7 to 10. Participants mentioned the peer components as part that they liked about the program. Participants disliked different aspects of the program, e.g. the exercises before the group walks.

## 4 Discussion

Non-complete case analyses indicated no significant changes in 6MWT performance, BMI, EQ-index, and EQ-VAS from week 1 (T0), to week 30 (T1), and to week 50 (T2). Complete case analyses showed a significant change in EQ-VAS over time (Appendix B). On average, the program was perceived well. However, since this pilot study consisted of a small convenience sample and the satisfaction survey yielded a small response, these results should be interpreted with caution.

One of the reasons for non-significant results in the outcome measures might be because of the small sample size in combination with individual differences between participants. For example, in the current study participants with complete data were more physically active before the start of the intervention compared to the participants with incomplete data. Additionally, professionals mentioned that bigger walking groups led to more differences in physical fitness levels (data not shown). This shows that individual differences in physical activity levels could be a key factor in the development and optimization of multicomponent interventions with a physical component.

Even though motivation was not explicitly measured during this study, it may provide an explanation for the dropout rates that were seen after week 30. Motivation plays a key role in a person's self-confidence and capability to maintain healthy choices, and in the efficacy of physical activity interventions (Hoogendoorn et al., 2019; Lakerveld et al., 2020). Complete case analyses showed a significant effect of time on EQ-VAS (see sensitivity analyses in Appendix B), which may indicate that only the most motivated participants completed all 50 weeks of the intervention. Another possibility could be that when professional guidance stopped after 30 weeks, groups fell apart and participants lost motivation.

Furthermore, adherence to physical activity might pose a problem for exercise focused interventions. Short-term interventions can be moderately successful under optimal circumstances, but poor adherence to exercise makes long-term effectiveness difficult (MacDonald et al., 2025). Conversely, complete case sensitivity analyses indicated improvements in QoL over time. This shows that those participants motivated to complete the intervention benefit more in terms of health outcomes. Overall, a more explicit focus on motivating participants to continue to walk with peers after the first 30 weeks might improve adherence and improve its effectiveness.

Multicomponent physical activity interventions show promise in encouraging health-promoting behaviour in various populations. For instance, in older adults multicomponent interventions are able to improve physical fitness, and mental health and well-being through group-based activities and social engagement (Chang et al., 2023; Mayela et al., 2023; Zhang et al., 2024). These examples and the current pilot study teach us that combining physical activity with environmental, behavioural, and educational strategies is beneficial; strategies tailored to target populations work best; and creating social and environmental support through stakeholders improves intervention participation and success.

A key strength of this pilot study is the multicomponent character of the intervention. Such an intervention has previously been found to be effective in people diagnosed with, or at risk of, T2DM (Hoogendoorn et al., 2019). Even though the outcome measures of the current study did not change significantly over time, there may have been changes in behaviour in other lifestyle factors addressed by the intervention: some of the participants' responses to the questionnaire indicated overall improvements in health and wellbeing. The last assessment was at the end of the intervention, so it is not possible to provide any information about the long-term effects of the intervention. This study was based on retrospective data from a completed pilot intervention, so no additional data or questionnaire responses were available other than the data that was presented in this study. Therefore, we do not know the reasons for subjects not responding to the questionnaire. The study was limited by the small sample size and dropout rates from T1 to T2, limiting generalizability of results. The participants in this study might be a more motivated group to begin with, and those who completed all measures might additionally be more motivated to complete the intervention compared to those dropping out.

Adherence to exercise and individual differences in physical fitness and motivation are some of the difficulties faced by walking interventions in T2DM. Recognizing these difficulties may help with the development and optimalization of future multicomponent walking interventions. One of the positive features of the current intervention is the peer aspect of walking in groups. Baseline testing can be used to determine groups, for instance based on baseline physical fitness or walking speed. Future research should investigate the effects of this 50-week multicomponent walking intervention with a bigger sample size. Furthermore, process evaluation after the intervention was limited to a small group of respondents, leaving valuable information untouched. Finally, because of the individual differences within the walking groups, future examinations should consider strategies at the beginning of the intervention that allow for walking groups of the same physical activity levels.

In conclusion, the current study elicited positive responses from individuals diagnosed with, or at risk of, T2DM. However, no significant changes over time in physical fitness, QoL, and BMI were found, possibly because of a small sample size and dropout rates. Multicomponent walking interventions with lifestyle coaching may have the potential to help in the prevention and management of T2DM. However, the current study showed that focusing on individual differences, motivation and adherence is needed.

## Declaration of generative AI in scientific writing

The authors declare no AI tools were used in the writing of this manuscript.

## Human rights

All procedures performed in studies involving human participants were in accordance with the ethical standards of the institutional and/or national research committee and with the 1964 Helsinki declaration and its later amendments or comparable ethical standards.

## CRediT authorship contribution statement

**Jesper Mulder:** Writing – review & editing, Writing – original draft, Methodology, Formal analysis. **Anne K. Smit:** Writing – review & editing, Methodology, Formal analysis. **Mirte Boelens:** Writing – review & editing, Writing – original draft, Methodology, Formal analysis. **Jessica C. Kiefte-de Jong:** Writing – review & editing, Methodology.

## Informed consent

Informed consent was obtained from all individual participants.

## Funding sources

This pilot study was conducted under the broader Diabetes Challenge project, which received ethical approval (METC number: 19–035) and received funding from the Bas van de Goor Foundation who is also funder of the Diabetes in Beweging intervention. The funder conceptualized, designed, and conducted the intervention. The funder did not have a role in data analysis, writing, or publication.

## Declaration of competing interest

The authors declare that they have no known competing financial interests or personal relationships that could have appeared to influence the work reported in this paper.

## Data Availability

De-identified data from this study will be made available by emailing the corresponding author and with approval from the Bas van der Goor Foundation.

## Appendix A Supplementary data

Supplementary material 1: Schematic representation of the 50-week walking intervention. Image 1

Supplementary material 2: Linear mixed model sensitivity analyses results of a six-minute walking test (6MWT), body mass index (BMI), quality of life index (EQ-index), and quality of life visual analogue scale (EQ-VAS) in Dutch adults with or at high risk of type 2 diabetes mellitus who had measures at two or more timepoints (Supplementary Table 1), and complete data (Supplementary Table 2). Image 2
