# Supplementary material for: A 50-week walking intervention for type 2 diabetes mellitus: A pilot study to improve fitness, BMI, and quality of life outcomes
Source: Prev Med Rep. 2025 Nov 1;60:103299. doi: 10.1016/j.pmedr.2025.103299 (PMC12639395; doi:10.1016/j.pmedr.2025.103299)
Supplement: Supplementary file 1 — Supplementary material 1: Schematic representation of the 50-week walking intervention. [file mmc1.docx]

**Appendix A: Schematic overview of the ‘Diabetes in Beweging’ intervention.**


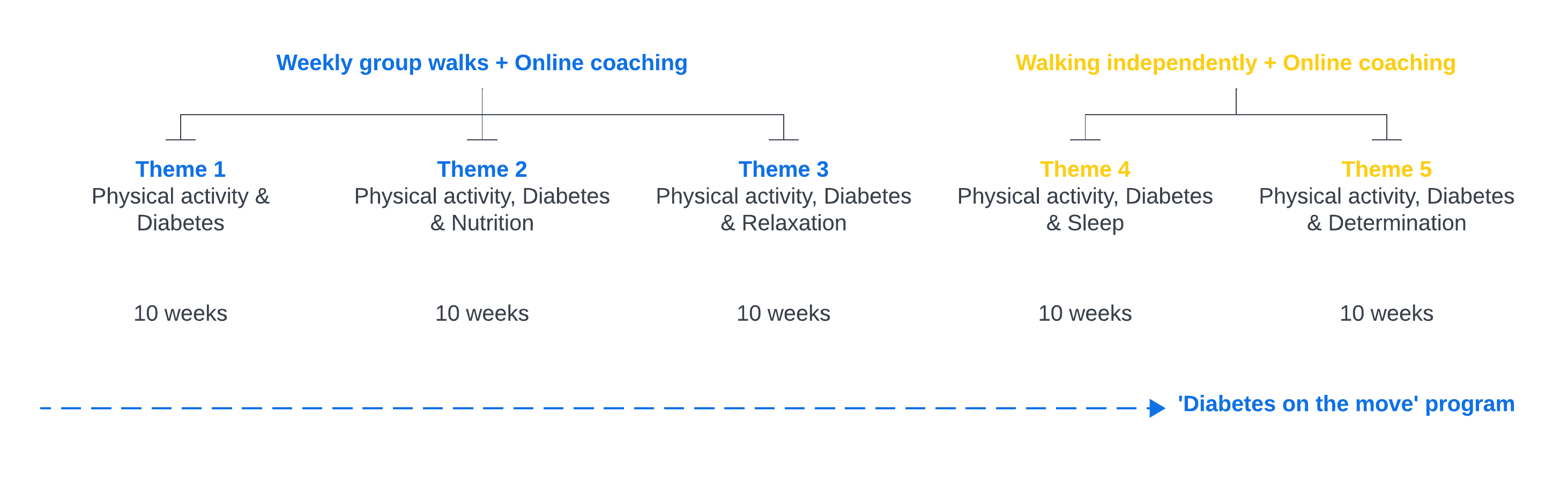


Figure 1. Schematic representation of the 50-week walking intervention.
