# Supplementary material for: A 50-week walking intervention for type 2 diabetes mellitus: A pilot study to improve fitness, BMI, and quality of life outcomes
Source: Prev Med Rep. 2025 Nov 1;60:103299. doi: 10.1016/j.pmedr.2025.103299 (PMC12639395; doi:10.1016/j.pmedr.2025.103299)
Supplement: Supplementary file 2 — Supplementary material 2: Linear mixed model sensitivity analyses results of a six-minute walking test (6MWT), body mass index (BMI), quality of life index (EQ-index), and quality of life visual analogue scale (EQ-VAS) in Dutch adults with or at high risk of type 2 diabetes mellitus who had measures at two or more timepoints (Supplementary Table 1), and complete data (Supplementary Table 2). [file mmc2.docx]

**Appendix B: Linear Mixed Model sensitivity analyses.**

Sensitivity analyses were performed using linear mixed models including 1) only those participants who had measures at two or more timepoints, and 2) only those participants who had complete data. To ensure no valuable data was lost, the presence of measures at two or more timepoints was checked separately per outcome measure (six-minute walking test, BMI, EQ-index, and EQ-VAS).

*Participants with measures at two or more timepoints*

The sensitivity analyses for the six-minute walking test consisted of 28 participants who had measures at two or more timepoints. The analyses indicated no significant changes over time, with the effect of time being non-significant (β = -5.866, CI = -17.630, 5.898).

The sensitivity analyses for BMI consisted of 31 participants who had measures at two or more timepoints. The analyses indicated no significant changes over time, with the effect of time being non-significant (β = -0.140, CI = -0.391, 0.111).

The sensitivity analyses for the EQ-index consisted of 30 participants who had measures at two or more timepoints. The analyses indicated no significant changes over time, with the effect of time being non-significant (β = -0.009, CI = -0.031, 0.012).

The sensitivity analyses for the EQ-VAS consisted of 30 participants who had measures at two or more timepoints. The analyses indicated no significant changes over time, with the effect of time being non-significant (β = 2.473, CI = -0.463, 5.410).

Appendix table 1. Linear mixed model analyses results of a six-minute walking test (6MWT), body mass index (BMI), quality of life index (EQ-index), and quality of life visual analogue scale (EQ-VAS) in 28 Dutch adults with or at high risk of type 2 diabetes mellitus who had measures at two or more timepoints.

|  | **β** | **CI Lower** | **CI Upper** |
| --- | --- | --- | --- |
| *6MWT* | | | |
| Time | -5.866 | -17.630 | 5.898 |
| Group | 1.711 | 0.167 | 3.255 |
| *BMI* | | | |
| Time | -0.140 | -0.391 | 0.111 |
| Group | 40.104 | 13.367 | 66.841 |
| *EQ-index* | | | |
| Time | -0.009 | -0.031 | 0.012 |
| Group | 4.768 | 1.242 | 8.293 |
| *EQ-VAS* | | | |
| Time | 2.473 | -0.463 | 5.410 |
| Group | 2.720 | 0.567 | 4.872 |

*Note: Linear mixed model analyses were not adjusted for covariates.*

*Participants with complete data*

The sensitivity analyses for the six-minute walking test consisted of 9 participants who had complete data. The analyses indicated no significant changes over time, with the effect of time being non-significant (β = 1.444, CI = -10.693, 13.582).

The sensitivity analyses for BMI consisted of 13 participants who had complete data. The analyses indicated no significant changes over time, with the effect of time being non-significant (β = 0.062, CI = -0.200, 0.323).

The sensitivity analyses for the EQ-index consisted of 11 participants who had complete data. The analyses indicated no significant changes over time, with the effect of time being non-significant (β = -0.023, CI = -0.054, 0.008).

The sensitivity analyses for the EQ-VAS consisted of 12 participants who had complete data. The analyses indicated a significant effect of time (β = 4.792, CI = 1.439, 8.145).

Appendix table 2. Linear mixed model analyses results of a six-minute walking test (6MWT), body mass index (BMI), quality of life index (EQ-index), and quality of life visual analogue scale (EQ-VAS) in 28 Dutch adults with or at high risk of type 2 diabetes mellitus who had complete data.

|  | **β** | **CI Lower** | **CI Upper** |
| --- | --- | --- | --- |
| *6MWT* | | | |
| Time | 1.444 | -10.693 | 13.582 |
| Group | 2.228 | -0.816 | 5.273 |
| *BMI* | | | |
| Time | 0.062 | -0.200 | 0.323 |
| Group | 51.123 | 1.034 | 101.211 |
| *EQ-index* | | | |
| Time | -0.023 | -0.054 | 0.008 |
| Group | 2.945 | -0.546 | 6.436 |
| *EQ-VAS* | | | |
| Time | 4.792 | 1.439 | 8.145 |
| Group | 1.941 | -0.370 | 4.252 |

*Note: Linear mixed model analyses were not adjusted for covariates.*
